# Optimizing Recruitment and Retention in Substance Use Disorder Research in Emergency Departments

**DOI:** 10.5811/westjem.2022.11.57179

**Published:** 2023-02-20

**Authors:** Lindsay M. Worth, Wendy Macias-Konstantopoulos, Lauren Moy, Harold I. Perl, Cameron Crandall, Roberta Chavez, Alyssa Forcehimes, Raul Mandler, Michael P. Bogenschutz

**Affiliations:** * University of New Mexico, Department of Psychiatric Research, Albuquerque, New Mexico; † Harvard Medical School, Massachusetts General Hospital, Department of Emergency Medicine, Boston, Massachusetts; ‡ New York University, New York, New York; § Independent Practice, Taos, New Mexico; ¶ The Change Companies, Carson City, Nevada; || University of New Mexico, Department of Emergency Medicine, Albuquerque, New Mexico; # University of New Mexico Center on Alcoholism, Substance Use Disorder and Addictions, Albuquerque, New Mexico; ** National Institute on Drug Abuse Clinical Trials Network, Bethesda, Maryland

## Abstract

**Introduction:**

Clinical trial recruitment and retention of individuals who use substances are challenging in any setting and can be particularly difficult in emergency department (ED) settings. This article discusses strategies for optimizing recruitment and retention in substance use research conducted in EDs.

**Methods:**

Screening, Motivational Assessment, Referral, and Treatment in Emergency Departments (SMART-ED) was a National Drug Abuse Treatment Clinical Trials Network (CTN) protocol designed to assess the impact of a brief intervention with individuals screening positive for moderate to severe problems related to use of non-alcohol, non-nicotine drugs. We implemented a multisite, randomized clinical trial at six academic EDs in the United States and leveraged a variety of methods to successfully recruit and retain study participants throughout the 12-month study course. Recruitment and retention success is attributed to appropriate site selection, leveraging technology, and gathering adequate contact information from participants at their initial study visit.

**Results:**

The SMART-ED recruited 1,285 adult ED patients and attained follow-up rates of 88%, 86%, and 81% at the 3-, 6-, and 12-month follow-up periods, respectively. Participant retention protocols and practices were key tools in this longitudinal study that required continuous monitoring, innovation, and adaptation to ensure strategies remained culturally sensitive and context appropriate through the duration of the study.

**Conclusion:**

Tailored strategies that consider the demographic characteristics and region of recruitment and retention are necessary for ED-based longitudinal studies involving patients with substance use disorders.

## INTRODUCTION

In the United States, the emergency department (ED) is an important healthcare access point, especially for underinsured and underserved populations with reduced access to other sources of care.^1^ In 2016 there were an estimated 145.6 million visits to non-federal hospital EDs in the United States,^2^ and a report published in 2010 by the Substance Abuse and Mental Health Services Administration found that almost half of all ED visits were related to drug misuse or dependence.^3^ Because EDs serve a high volume of individuals with substance use disorders, ED visits present opportunities for screening, brief intervention, and referral to treatment (SBIRT).^4^

There are some distinctive barriers inherent in recruiting individuals with substance use disorders. The rate of recruitment in clinical trials for addiction research is linked to location and size of the recruitment site, the target population, the inclusion and exclusion criteria, and the perceived benefit to the participant of the treatment offered.^5^ The natural inclination for individuals to understate or hide highly stigmatized behaviors presents obstacles in both recruitment and data quality.^6^–^10^ Additionally, patients may decline to participate because of a number of reasons including not feeling well, lack of interest, concerns about confidentiality, and the time-consuming nature of the study.^11^–^13^ Obtaining a representative sample of the population of interest and agreement rates of 70% or more support generalizability of that population.^14^–^16^

Participant compensation is another important consideration. Participants may perceive low compensation as patronizing, while excessive payment can compromise voluntary consent.^17^ Yet even though it is important to establish appropriate compensation for participation,^16^ it is not the most important factor in securing enrollment.^18^–^19^ Study staff flexibility (eg, taking breaks from study assessments for medical interventions) and rapport-building (eg, expressing compassion) are considered two of the most important determinants in successful recruitment for ED-based clinical trials.^19^–^25^

In medical settings, collecting data from patients with electronic devices, such as tablet or laptop computers, has proven to be an acceptable^26^ and time-saving^27^ method for gathering information. Allowing participants to complete behavior assessments electronically minimizes feelings of embarrassment and judgment and improves a sense of privacy compared to study staff interview methods.^28^–^30^ Several studies suggest that electronic screening outperforms verbal screening in detecting adversity across a spectrum of potentially sensitive topics among ED patient populations. 28–30

In addition to its role in data collection and data quality, technology has also proven useful with participant tracking in longitudinal studies. Both free and fee-based online search tools, online public records, and social networking sites are useful for locating participants.^21^,^31^ Longitudinal ED-based research requires a variety of retention strategies including collecting adequate participant contact information; making repeated contact attempts for follow-up visit completion including in-person, phone calls, mailed letters, and web-based strategies; and allowing for flexibility in the location of follow-up completion.^32^

Although extensive research has been done on SBIRT in alcohol use disorder, much less SBIRT research has been done with other substance use disorders.^1^,^33^–^34^ To address this gap, we conducted a multisite trial “Screening, Motivational Assessment, Referral, and Treatment in Emergency Departments (SMART-ED)” through the National Institute on Drug Abuse Clinical Trials Network (NIDA CTN) to compare the effectiveness of 1) a brief motivational interviewing^36^ intervention; 2) screening, assessment and referral; and 3) minimal screening only in an ED sample of patients with probable SUD. Conducting multisite clinical trials with complex behavioral interventions in the ED presents numerous challenges in recruitment and retention of participants. We describe our recruitment and retention experiences and the lessons learned while conducting this ED-based, multisite SBIRT study.

**Population Health Research Capsule**

What do we already know about this issue?

*Clinical trial recruitment and retention of individuals who use substances are challenging in any setting and can be particularly difficult in ED settings*.

What was the research question?

*How can we maximize recruitment and retention of individuals who use substances who are patients in the ED?*

What was the major finding of the study?

*Recruitment goals were met: 1,285 were enrolled in the study and the 3-, 6-, and 12-month retention rates for this study were 89%, 86%, and 81%, respectively*.

How does this improve population health?

*Successful recruitment and retention allow for a better understanding of how an intervention in the ED impacts current and future substance use*.

## METHODS

Recruitment and initial baseline assessment for the SMART-ED study took place between October 2010–February 2012 in six urban academic EDs in the US, each of which partnered with a node of the NIDA CTN (**Trial Registration** www.clinicaltrials.gov Identifier: NCT01207791).^34^ Three sites were on the East Coast and one in each of the Midwest, South, and Southwest regions (Table 1).^34^ Site selection criteria for this study included the following: EDs that collectively had a patient population broadly representative of the US population; an adequate number of ED patients with SUD; ED research experience and infrastructure; access to a referral network for specialty addiction treatment; and EDs with the sufficient staff and willingness to participate and implement the study protocol.

For this study our goal was to enroll 1,285 participants across sites over a nine-month period and complete follow-up visits at three, six, and 12 months post baseline.

We used tablet computers for a number of project activities: 1) to screen and collect data; 2) access the electronic health record; and 3) collect participant contact information. In addition to eliminating the need for paper forms, tablet computers allowed study staff to receive immediate notification of participant eligibility and group randomization. Study staff approached potentially eligible patients after triage. The ED tracking boards helped to locate patients. Table 2 lists the complete inclusion and exclusion criteria. Every effort was made to meet with patients in a private room, although this was often a challenge. At one site, study staff placed a partition in the corner of the waiting room and used this space to screen patients for the study.

Once they were enrolled, we collected participant contact information including 1) residential and mailing address, 2) phone number(s), 3) email address, 4) Social Security number, 5) place of employment, and 6) contact information for two “locators” (ie, persons who would know how to contact the participants during the course of the study). If they were not able to provide sufficient contact information, they were not eligible to participate (Table 2). Although Social Security numbers were gathered as a part of the form used for this study, they were not used to track participants in this study. Participants were randomly assigned to one of three cohorts: 1) brief motivational interviewing intervention; 2) screening assessment and referral; or 3) minimal screening only.

Compensation for completing the baseline and each follow-up assessment was $50 and $75, respectively. Baseline assessments took between 60–120 minutes, and follow-up assessments ranged between 90–210 minutes to complete. At a separate location from the ED, staff (who were blinded to treatment assignment) conducted follow-up assessments. Appointment cards, maps, and study contact information were provided at the initial ED baseline visit, and reminder calls were made prior to each follow-up visit.

When a participant attended their follow-up study visit, staff were required to review and update all participant contact information. If a participant did not attend their follow-up visit, staff would, in order, do the following: attempt to reach the participant by varying times of call attempts; send email and text message; mail a letter to the participant; and contact the participant’s locators. If staff were unsuccessful in reaching the participant, they would conduct an internet search to try to obtain more current contact information. At one site, follow-up staff attempted to locate the participant in person at their home address. Across and within sites, there did not appear to be a single approach to locating participants and scheduling follow-ups that emerged as superior to another approach.

Follow-up staff documented all contact attempts, regardless of success, in the “Contact Log,” which included date and time of attempted contact and a description and result of the attempt (Figure). Documentation allowed staff to see what type of contacts had already been attempted. Unsuccessful tracking methods and bothering a participant or locator who may have been recently contacted were not repeated. In addition to using the participant contact information provided to help locate participants for follow up, other accommodations such as meeting at a more convenient location (depending on institutional review board [IRB] rules), varying times to meet, or a phone option for conducting follow-up were offered. Study staff also emphasized that participation in the study was voluntary.

Participant incarceration is an expected occurrence that poses challenges to completing follow-up. In anticipation of this reality, we obtained Office for Human Research Protections approval to conduct follow-up visits with participants who became incarcerated after enrollment, and the SMART-ED study sites pursued IRB approval. Ultimately, the ability of the study staff to follow up with the participant depended upon the study site’s IRB regulations, type of consent obtained from the participant, and the rules of the confining correctional facility.

Another challenge to retention was the occasional participant request for withdrawal when contacted to schedule follow-up visit appointments. In such cases, we honored the request and mailed the participant a letter confirming their decision to withdraw, providing the study’s contact information, reviewing the benefits of participation, and inviting them to contact the study should they change their mind.

Ongoing study staff training occurred throughout the study, emphasizing the importance of 1) recruitment study staff approaching all potentially eligible patients (post-triage) without regard to diagnosis, thus, improving the representativeness of the sample; and 2) follow-up study staff reviewing the methods for contacting participants. Additionally, weekly recruitment and retention calls with all sites provided a forum to discuss any recruitment and retention issues, clarify procedures, and troubleshoot unanticipated problems.

Over the course of the study, we made several adjustments to improve participant retention. These adjustments included decreasing assessment time at follow-up (ie, fewer assessments administered at follow-up), expanding the time windows for completing assessments, and providing incentive compensation to study staff at sites who achieved an 85% follow-up rate or higher. The original four-week time window for completing follow-up assessment (two weeks before and two weeks after the ideal follow-up date) was opened to allow a participant six weeks to complete follow-up visit (two weeks prior and four weeks post the ideal follow-up date). For example, if someone’s follow-up was due on February 14, they could be seen as early as February 1 or as late as March 14 for their follow-up.

This expanded follow-up time window offered participants increased flexibility and convenience when scheduling their visits without compromising follow-up data integrity. The incentive compensation offered to study staff who achieved 85% follow-up rates or higher was in the form of a $5 gift card to a coffee shop for each staff member involved in follow-ups at that site. This amount was felt to increase team motivation and promote friendly competition across the study sites to complete follow-up visits with participants, without encouraging coercive practices or dishonest reporting.

## RESULTS

Sites recruited participants for this study over a 16-month period during which a total of 20,762 patients were approached for an initial screening. Of those, 15,224 (73.3%) patients gave verbal consent to anonymously complete an electronic screening questionnaire to determine eligibility. Based on eligibility, willingness to participate, and ability to continue, 1,285 patients were enrolled in this study, on target with recruitment projections. We excluded patients who had an incomplete screen (252), fell below the cutoff score of the Drug Abuse Screening Test for problematic drug use (12,888), failed to meet inclusion criteria (64), did not complete consent (725), or withdrew prior to randomization (10). Table 3 provides an overview of study participant characteristics.

Tracking and retention occurred over a 29-month period during which staff completed 3,179 follow-up assessments. The 3-, 6-, and 12-month retention rates for this study were 89%, 86%, and 81%, respectively. Follow-up rates did not vary by group assignment (Table 4). Aside from being unreachable for follow-up, other reasons for missed follow-ups included incarceration, study withdrawal, and death.

As many as 70 contact attempts were made for a few participants before they completed a follow-up. Follow-up staff made on average 26 contact attempts per participant to schedule a follow-up appointment. Contact attempts included making phone calls to participants and locators; texting; sending letters and email messages; conducting online searches to include searching obituaries and incarceration websites; visiting the participant’s home; and on occasion, if approved by the local IRB, sending private messages on Facebook. Phone calls were the most common method used to contact a participant. Varying the time of calling and the days when a participant was called increased the success of reaching and scheduling follow-ups with participants.

Of the 3,176 follow-up assessments completed, 2,918 (91.8%) were in person and 261 (8.2%) over the telephone. We identified 64 participants incarcerated at some point in the follow-up period. Comprehensive study results can be found in the author MB’s 2014 primary outcomes paper.^35^

## DISCUSSION

The population in this study included ED patients with SUD. Retention at the three-month follow-up was 89% and remained above 80% for subsequent follow-ups. Site selection based on predetermined criteria, inclusion and exclusion criteria that included criteria that increased the likelihood of successful follow-up with participants, adequate compensation for study visits, ongoing training and monitoring of recruitment and retention efforts, effective use of technology (eg, tablet computers), and flexibility in enrollment and conducting follow-up assessments were factors considered to support successful recruitment and retention of participants. Urban sites with a large ED census of patients and availability of substance use treatment programs were also key factors in site selection for this study (Table 1). Additionally, patient population characteristics were considered for generalizability of the study (Table 3).

We chose certain inclusion and exclusion criteria to support successful follow-up with participants (Table 2). Criteria for inclusion that contributed to ease of contacting participants for follow-up included access to a phone, residence within 50 miles from the location of follow-up visits, and ability to provide sufficient contact information (required to provide at least two reliable locators). “Locators” are individuals who may have contact information for the participant if the follow-up staff are not able to reach the participant. Although the same recruitment and retention guidelines were used across study sites, the success of using these guidelines varied; methods that worked well at one site were not always effective across sites. It was important to allow sites to adapt general study guidelines that best suited their population and environment.

Staff flexibility at enrollment (eg, meeting patients when they felt well enough to complete assessments and were not busy with medical care) and follow-up (eg, completing follow-ups by phone or in the community and when convenient to the participant), was the single most likely factor to have mediated the success in recruitment and retention. We did not gather data on the participant’s opinion of using tablet computers, but it is hoped that this minimized any feelings of embarrassment or perceived stigmatization in reporting sensitive drug use information. Similarly, compensation is presumed to have been acceptable as there were no complaints about compensation being too little or too much over the course of the study.

The average number of contact attempts was 26 and ranged up to 70 to reach a participant for a follow-up visit. Most commonly, participants or their locators were reached by phone or via letters sent, but conducting online searches and using social media (ie, Facebook) to connect with participants were important access points as well. Both the amount of time and effort this intense level of follow-up entailed and the potential for participants to feel harassed or coerced to participate must be seriously considered. To ensure participants do not feel harassed or coerced, it is important to emphasize that participation is voluntary. Additionally, documentation of contact attempts ensures that participants who have refused to participate are not contacted again and contact methods that have been unsuccessful are not repeated. The level of effort to contact participants is time-consuming, and it is important to appropriately plan for this. Likewise, thoughtful and strategic outreach to enrolled patients requires careful internal documentation and communication within the follow-up team, also requiring time and effort.

For future research in EDs we would recommend using wireless internet data cards rather than relying on wireless connections to the ED network. Losing internet connection became a point of frustration for both participants and enrollment study staff conducting interviews as they would sometimes lose data and be forced to repeat parts of the baseline assessment. A wireless internet data card allows users to access online information anytime and anywhere without getting disconnected from the network.

Obtaining participant consent upfront for texting, emailing, and searching for participants through publicly available data including social media networks such as Facebook is recommended. We implemented this midway through the study, and some study sites had difficulty in gaining permission from their IRBs to use these resources without participant consent. We would also recommend seeking IRB approval and participant consent to continue working with enrolled study participants who might become incarcerated during the study.

## LIMITATIONS

Allowing follow-ups to occur outside the target follow-up date may have inflated retention results slightly, but because these windows were well-defined and narrow, the impact on data was minimal and we feel the benefit to follow-up rates and data collection justifies the approach.

## CONCLUSION

Consistent with the research, we found that recruitment of ED patients with substance use disorder and retention of these participants in a longitudinal study required a multifaceted process. We found that certain methods for recruitment and retention were useful across sites (eg, exclusion criteria, consent for contact through social media, IRB approval of procedures to retain incarcerated patients), but it was also important to consider the location of a study site in tailoring and developing additional strategies.”

## Figures and Tables

**Figure:**
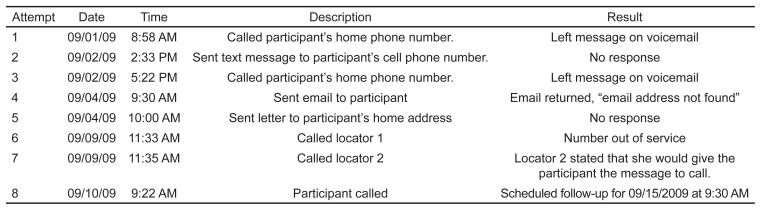
Sample contact log.

**Table 1:** Site characteristics.

Site regions	Trauma center designation	Annual ED visits	Urban vs rural (state)	
East Coast site 1	Level I	>100,000	Urban (MA)	Major teaching hospital (AMC)*
East Coast site 2	Level I	96,000	Urban (NY)	Major teaching hospital (AMC)*
East Coast site 3	Level I	54,000	Urban (WV)	Major teaching hospital (AMC)*
Midwest site	Level I	>75,000	Urban (OH)	Major teaching hospital (AMC)*
South site	Level I	120,000	Urban (FL)	Major teaching hospital (AMC)*
Southwest site	Level I	>80,000	Urban (NM)	Major teaching hospital (AMC)*

* Major teaching hospital or academic medical center is defined as a teaching hospital with an affiliated medical school.

*ED*, emergency department; *MA*, Massachusetts; *NY*, New York; *WV*, West Virginia; *OH*, Ohio; *FL*, Florida; *NM*, New Mexico; *AMC*, Academic Medical Center

**Table 2:** Study inclusion and exclusion criteria.

Inclusion criteria
1. Registration as a patient in the ED during study screening hours
2. Positive screen (>3) for problematic use of a non-alcohol, non-nicotine drug based on the Drug Abuse Screening Test
3. At least one day of problematic drug use (excluding alcohol or nicotine) in the past 30 days
4. Age 18 years or older
5. Adequate English proficiency
6. Ability to provide informed consent
7. Access to phone (for booster sessions)
Exclusion criteria
1. Inability to participate due to emergency treatment
2. Significant impairment of cognition or judgment rendering the person incapable of informed consent (eg, traumatic brain injury, delirium, intoxication)
3. Status as a prisoner or in police custody at the time of treatment
4. Current engagement in addiction treatment
5. Residence more than 50 miles from the location of follow-up visits
6. Inability to provide sufficient contact information (must provide at least 2 reliable locators)
7. Prior participation in the current study

**Table 3:** Study participant characteristics.

Characteristic	Total [N (%) or mean (SD)]
Gender	
Male	898 (70)
Female	387 (30)
Mean Age, mean (SD)	36 (12)
Ethnicity	
Hispanic or Latino	305 (24)
Not Hispanic or Latino	971 (76)
Chose not to answer	9 (1)
Race	
American Indian or Alaska Native	24 (2)
Asian	8 (1)
Black or African American	440 (34)
Native Hawaiian or Pacific Islander	5 (0)
White	641 (50)
Other	66 (5)
Multiracial	63 (5)
Unknown	15 (1)
Chose not to answer	23 (2)
Education completed	
1–11y	408 (32)
GED/12y	417 (32)
Some college	338 (26)
College degree	94 (7)
Some graduate	10 (1)
Graduate degree	16 (1)
Postgraduate degree	2 (0)
Marital status	
Married	122 (9)
Remarried	1 (0)
Widowed	27 (2)
Separated	86 (7)
Divorced	158 (12)
Never married	776 (60)
Cohabitating, not married	115 (9)
Employment in past 30 days	
Full-time	244 (19)
Part-time	209 (16)
Student	84 (7)
In controlled environment	3 (0)
Retired/disability	187 (15)
Service	0
Homemaker	12 (1)
Unemployed	546 (42)
Annual household income	
$0–$15,000	804 (63)
$15,001–$30,000	180 (14)
$30,001–$50,000	80 (6)
$50,001–$75,000	36 (3)
$75,001–$100,000	22 (2)
>100,000	13 (1)
Declined to answer	150 (12)
Primary substance	
Cannabis	567 (44)
Cocaine	349 (27)
“Street” opioids	218 (17)
Prescription opioids	69 (5)
Methamphetamines	49 (4)
Sedatives and sleeping pills	20 (2)
Hallucinogens	9 (1)
Prescription stimulants	3(0)

*GED*, General Equivalency Diploma.

**Table 4:** Follow-up rates by group assignment.

	Brief motivational interviewing intervention (N=427)	Screening, assessment and referral (N=427)	Minimal screening (N=431)
		N (%)	
Completed 3-month follow-up	375 (88)	382 (90)	382 (89)
Completed 6-month follow-up	362 (85)	370 (87)	375 (87)
Completed 12-month follow-up	338 (79)	348 (82)	357 (83)
